# The temporal dynamics of task processing and choice in a novel multitasking paradigm

**DOI:** 10.1007/s00426-024-01971-8

**Published:** 2024-05-11

**Authors:** Victor Mittelstädt, Ian Grant Mackenzie, Sebastian Heins, Jeff Miller

**Affiliations:** 1 Department of Psychology, University of Tübingen, Schleichstraße 4, 72076 Tübingen, Germany; 2 University of Otago, Dunedin, New Zealand

## Abstract

This study investigated the temporal dynamics of task performance and voluntary task choice within a multitasking paradigm in which the task-related processing outcomes themselves determined the to-be-performed task. In the novel forced-no-go trials, the stimulus for one task required an overt response, but the stimulus for the other task was associated with a no-go response. Task performance results showed that participants often processed the no-go task’s stimulus before switching to the go-task. Dual-task interference effects and switch costs indicated various forms of multitasking interference, with their underlying causes appearing to overlap, as engagement in parallel processing seemed to be limited by switch-related reconfiguration processes. Intermixing free-choice trials, where both stimuli were associated with overt responses, revealed costs associated with switching between processing modes, providing new evidence that the distinctions between free and forced task goals stem from differences in their internal representations rather than alterations in processing due to different presentations in the environment. Task choice results align with this perspective, demonstrating a preference for repeating a free- over a forced-choice task. Furthermore, these free-choice results illuminate the interplay of cognitive (task-repetition bias) and environmental constraints (first-task bias) in shaping task choices: It appears that task-specific information increases goal activations for both task goals concurrently, with participants favoring central processing of the second- over the first-presented task to optimize their behavior when shorter central processing is required (task repetition). Overall, this study offers new insights into the dynamics of task processing and choice in environments requiring the balance of multiple tasks.

## Introduction

In our modern society, we are constantly confronted with diverse information associated with various tasks, leading us to frequently engage in sequential multitasking, commonly known as task switching (e.g., Kiesel et al., 2010, Meiran, 2008, Jersild, 1927, Vandierendonck et al., 2010). As described below, various task-switching paradigms have been developed for studying the mechanisms and consequences of such switching in controlled laboratory settings (e.g., Kiesel et al., 2010, Altmann, 2004, Rogers &Monsell, 1995, Meiran et al., 2000, Kleinsorge et al., 2001, Waszak et al., 2003). The results of these paradigms have clearly demonstrated that task switching is generally slower, and each of the specific paradigms has contributed much information about the causes of these switch costs. In essence, these switch costs reflect capacity limitations in reallocating cognitive resources between different tasks across consecutive trials. These limitations are typically attributed to specific cognitive constraints imposed by overcoming passive interference from the previously applied task goal and/or the active reconfiguration required to retrieve a new task goal (e.g., Koch et al., 2018, Verschooren et al., 2019, Meiran, 2008).

However, switch costs reflect only one aspect of multitasking. Specifically, capacity limitations are also present when we engage in concurrent multitasking, commonly known as dual-tasking (e.g., Koch et al., 2018, Pashler, 1994), with increased costs observed when the processing of two tasks more strongly overlaps within trials. These costs are typically attributed to structural limitations that allow only serial access to limited cognitive resources (e.g., Pashler, 1994, bottleneck models) or to strategic sharing of these resources to allow parallel task processing (e.g., Navon & Miller, 2002, resource-sharing models). Although theoretical suggestions exist on how to integrate these different multitasking phenomena within common frameworks (e.g., Hazeltine and Schumacher, 2016, Logan & Gordon, 2001, Koch et al., 2018), they are still primarily studied in separate paradigms.

In addition to task performance, researchers are interested in task choice behavior when faced with multiple tasks, as people can typically voluntarily decide which task to perform at a given time. (e.g., Mittelstädt et al., 2019, Arrington & Logan, 2005, Braem, 2017, Lien & Ruthruff, 2008, Brosowsky & Egner, 2021, Vandierendoncket al., 2012, Dreisbach & Jurczyk, 2022, Kang & Chiu, 2021, Brüning et al., 2021). Although there are recent suggestions that link task performance to task choice behavior to explain why people avoid cognitive constraints when switching tasks by consistently preferring to repeat tasks (cf. Mittelstädtet al., 2019, e.g., optimizing task performance), task choice is still often studied independently from task performance.

Drawing inspiration from previous paradigms (Fröber & Dreisbach, 2017; Miller & Durst, 2014), the main purpose of the present study was to introduce a novel “choice/no-go” multitasking paradigm that allows measuring different multitasking phenomena (switch costs, dual-task costs, task choice repetition bias) within one setting to provide further insights about the nature and generality of the mechanisms underlying task performance and task choice behavior when multitasking.

### Previous task switching paradigms

In many task-switching paradigms, participants must perform a sequence of trials in which the to-be-performed task is determined by the experimenter. The to-be-performed task may be indicated by the stimulus display; for example, the display might only include the stimulus for one task, or it might include a separate cue—not part of either task—indicating which task must be performed in the current trial (e.g., Schneider & Logan, 2005; 2011, Koch & Allport 2006, Gade & Koch, 2007) Alternatively, participants might be required to perform the tasks in a pre-determined, predictable sequence (e.g., Bratzke & Bryce, 2019, Rogers & Monsell 1995, Yeung & Monsell, 2003, alternating runs). Furthermore, the stimulus display in each trial might or might not also remind the participant of which task is required (Kleinsorge & Gajewski, 2008, Koch, 2003, Dreisbach & Haider, 2006, e.g., via a single stimulus or an external cue;). Task-switching may also be studied in paradigms where the sequence of tasks is determined by the participant—that is, in voluntary task-switching paradigms—in which participants are allowed to choose freely which task to perform in each trial (e.g., Mittelstädtet al., 2021, Arrington & Logan, 2005, Mayr & Bell, 2006). Furthermore, these two paradigm options can be combined in hybrid paradigms, where trials with experimenter-determined tasks are intermixed with voluntary choice trials (e.g., Mendl & Dreisbach, 2022, Jurczyk et al., 2019, Mittelstädtetal., 2023). For example, a hybrid paradigm might include a mix of trials with stimuli for one or both tasks, with participants allowed to choose the task freely when both stimuli are present. Another option is to present both stimuli in all trials but also present an external cue indicating whether participants should perform one task, should perform the other task, or should decide for themselves which task to perform (e.g., Qiao et al., 2023).

Using an experimenter-determined sequence of tasks has the advantage that it provides precise control over the sequence of task repetitions and switches, but existing methods for controlling the task sequence involve some limitations and complications. Presenting single stimuli, for example, is an extreme simplification of real-world multi-tasking situations where multiple stimuli are almost always present, and it trivializes the process of task selection. Presenting multiple stimuli with cues to indicate the relevant task seems more realistic, and yet, in this case, the processing of the cue itself is implicitly required as an additional task. This is problematic not only because the cue is somewhat distracting from the main tasks and adds to the overall processing load (e.g., Lavie et al., 2004), but also because it can be difficult to separate effects associated with cue processing from effects associated with task processing (e.g., Qiao et al., 2023). Indeed, in such cuing paradigms every trial is, in some sense, a switch from the cue processing task to one of the other tasks. Similarly, when the different tasks have to be performed in a pre-specified, predictable sequence, an additional implicit memory-based task is usually required for participants to keep track of their position in the sequence. If the sequence is predictable and cues are also used, then it may be unclear which of these implicit additional task requirements participants are performing, and the additional task (i.e., cue-based processing and/or memory-based sequence tracking) might vary from participant to participant or even trial to trial.

### The “choice/no-go” multitasking paradigm

The main purpose of the present study was to introduce and investigate a new “choice/no-go” paradigm in which the series of to-be-performed tasks is determined by the outcome of the task-related processing itself. As described in more detail below, the new paradigm combines the most desirable features of previous task-switching paradigms while avoiding some of the problematic features. Moreover, it incorporates properties of dual-tasking paradigms, as well as free-choice trials, making it possible to investigate various forms of interference in task performance and task choice behavior in multitasking within a single paradigm. Thus, on a broader level, the development of the paradigm was motivated by investigating whether it is generally possible to measure different multitasking effects within a single paradigm—a potential methodological advancement that seems helpful in working towards more overarching multitasking theories (e.g., Hazeltine & Schumacher, 2016, Logan & Gordon, 2001).

**Fig. 1:**
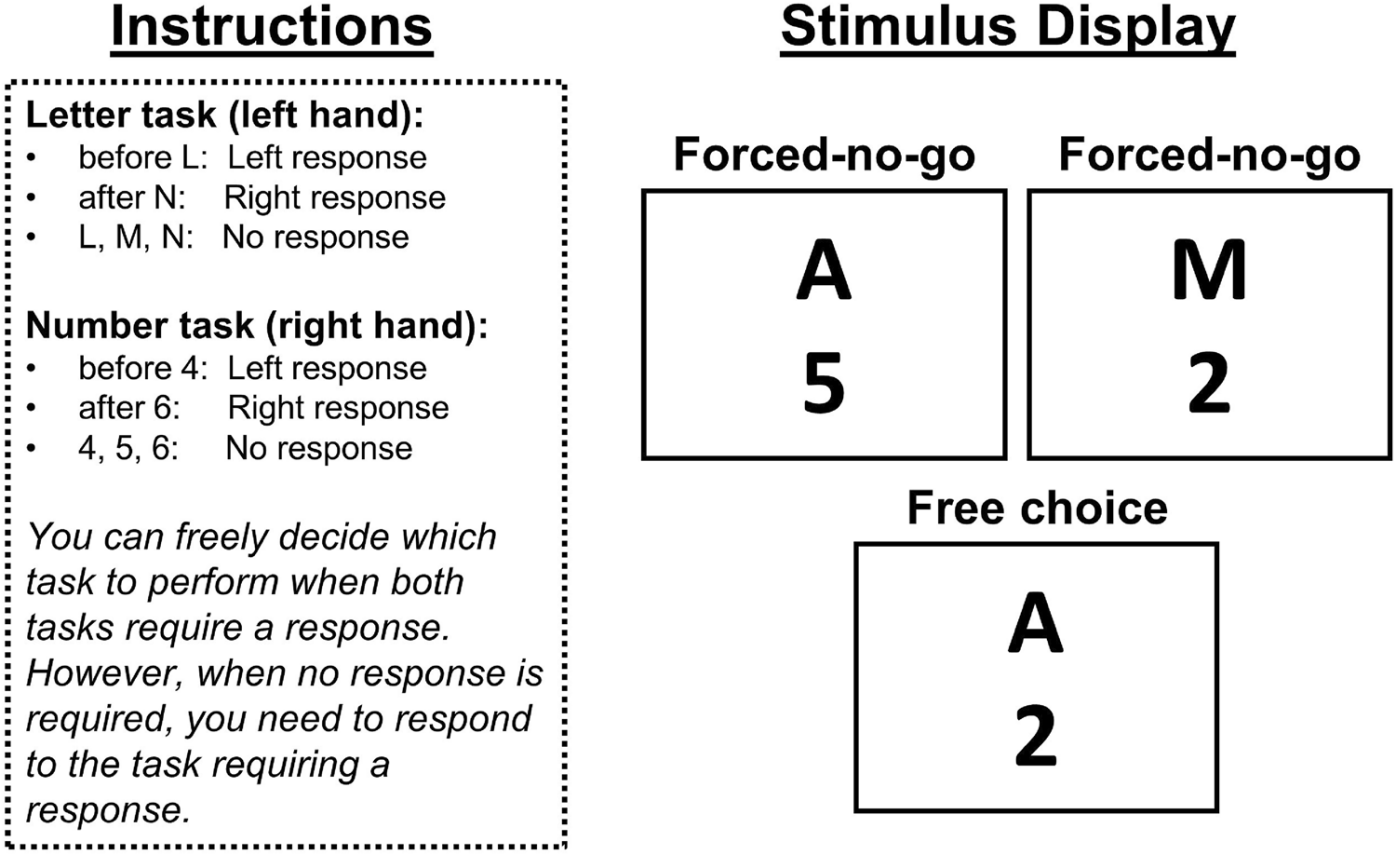
Central instructions and possible stimulus displays in Experiment 1 and Experiment 2. Note that in the actual experiment there were also fixation displays, feedback displays, intertrial intervals and when two stimuli were presented, they were separated by a random stimulus onset asynchrony of 50 ms versus 300 ms. In Experiment 1, we additionally implemented trials in which only a single stimulus was presented (forced-single)

Specifically, in each trial of the new paradigm the stimuli for two separate two-choice/no-go tasks are presented (cf. Fig. 1). In every trial the stimulus for one task is assigned to one of the choice-task responses, and the participant must perform this task. In that same trial the stimulus for the other task is assigned to the no-go response, so no overt response is required for the other task. Thus, the sequence of to-be-performed tasks within the new paradigm is determined entirely by the experimenter-controlled sequence of go and no-go stimulus pairs for the two tasks. However, because no-go responses also need to be selected (e.g., Logan et al., 2014, Mittelstädt et al., 2022a, Wühr & Heuer, 2020), task-specific response selection processes are required to determine a no-go response. To study effects on voluntary task choices, we used a hybrid variant of our two-choice/no-go paradigm. Specifically, in some trials the stimuli for both tasks were associated with two-choice responses, and participants were instructed that they could freely choose which response to make in these trials. Although several other studies have previously implemented no-go trials in task-switching paradigms (e.g., Koch & Philipp, 2005, Scheil & Kleinsorge, 2022), an additional cue was used in these studies to signal whether a response was needed for the presented task stimulus or not. In contrast, in our study, a no-go task stimulus determined whether a response to another task was required, so the required task was determined by task processing itself.

This new hybrid choice/no-go task-switching paradigm is of interest for several reasons. From an applied perspective, certain real-world task-switching environments seem more similar to this paradigm than to the cueing and predictable-sequence paradigms. For example, consider a situation where you are required to read and respond to two different emails. While reading one email, you might realize that no response is necessary and thus switch to the task of reading the other email. Alternatively, imagine interrupting your work to go to the office kitchen and brew a pot of coffee, only to discover that a colleague has already made the coffee, so you might switch to the task of organizing kitchen supplies. Moreover, because in many situations people have to decide between multiple tasks with each requiring a response, it seemed useful to consider free choice trials in this paradigm.

One major methodological advantage is that stimuli for two tasks can be presented in every trial not only in the free-choice condition but also in the two-choice/no-go forced condition, so that all responses are chosen in a multi-tasking context. As no extraneous cues are required to tell participants which task should be performed in a trial (e.g., task cue or predictable sequences), participants can focus entirely on the tasks that must be performed. Thus, from a theoretical perspective it is of interest to see whether previous patterns of task-switching results obtained with task cues and predictable sequences will generalize to this somewhat different multi-tasking context in the forced condition (e.g., switch costs). Moreover, as participants do not know in advance which task (if any) will be a no-go, they must be generally prepared for both tasks and may process both tasks in forced-no-go trials to determine the task requiring a response, allowing for the possibility of obtaining a pattern of results that may also capture concurrent multi-tasking (e.g., dual-task costs). Finally, by intermixing free-choice trials with the present novel forced-no-go trials, it is possible in addition not only to investigate how (multi-) task performance is linked to task choice behavior, but also to more fairly investigate potential differences in how we internally represent free- versus forced-choice tasks, as in both conditions two stimuli are presented.

### The present experiments: potential effects and their underlying mechanisms

Overall, the goal of the present two experiments was to introduce the hybrid “choice/no-go” paradigm by investigating potential effects on task performance and task choice to obtain a more connected picture of the mechanisms underlying different aspects of multitasking. For this purpose, it seemed especially useful to study the detailed temporal dynamics of task switching in this paradigm, because in this case the RTs would reflect *only* task-related processing and switching, with no additional time required for cue processing or memory retrieval. Thus, to get a better picture of these dynamics, we manipulated the stimulus onset asynchrony (SOA) between task-specific stimuli in each trial.

#### Task performance in forced-no-go trials

As the stimulus order and SOA (short, long) varied randomly across trials, it was unpredictable for participants whether the first or second stimulus was associated with a go versus no-go response (stimulus order S_1_-no-go and S_2_-go vs. S_1_-go and S_2_-no-go). Assuming that participants would usually start processing S_1_ first, a response to S_2_-go should be particularly delayed at short compared to long SOA. If so, this would support the idea of capacity-limited no-go processing and provide evidence of dual-task costs, as this essentially resembles a PRP-like effect (i.e., a decrease in RT to the second of two task-relevant stimuli) that is typically only observed in dual-tasking paradigms with predetermined task order (e.g., Miller & Durst, 2015, Mittelstädt & Miller, 2017). In addition, it seemed reasonable to expect that responses would be slower when the performed task switched rather than repeated, assuming that switching tasks is costly even without cue processing or memory retrieval of task sequences. If we indeed observe both types of multitasking interference, it remains to be seen whether they interact as one might assume if the underlying causes overlap. Finally, as the forced-choice trials can be preceded by either another forced-choice trial or a free-choice trial, we will investigate whether switching between these two processing modes is itself costly, as one might assume if the internal representations between free- and forced-task goals differ.

#### Task choice in free choice trials

In general, it has been suggested that people select the most active task goal representations, and that task choice can be linked to task performance. Thus, the effects on task choices may at least partially align with the ones found on task performance. Assuming again that participants will usually start processing S_1_ first, participants may be generally biased to choose S_1_-go over S_2_-go if the earlier task-specific information increases and speeds the activation of its associated task goal. Moreover, as the previously performed task is usually the most active one and because switching tasks is costly, participants may be generally biased to repeat a task. Finally, it remains to be seen how voluntary task choices would be influenced by the processing mode and outcomes in preceding trials. For example, participants may particularly avoid switching away from a freely-chosen compared to a forced-chosen task if a freely-chosen goal is more strongly activated. Moreover, working on a task that required a no-go response might be perceived as wasted effort, which might tend to make participants avoid that task in the following trial.

## Experiment 1

The basic tasks used in this experiment (as well as the next) were letter and number categorization tasks. For the letter task, participants had to respond when the letter was before L or after N in the alphabet (A, B, C, X, Y, Z as go stimuli) and not respond when the letter was L, M or N (i.e., no-go stimuli). For the number task, participants had to respond when the number was before 4 or after 6 (1, 2, 3, 7, 8, 9 as go stimuli) and not respond when the number was 4, 5 or 6 (i.e., no-go stimuli). In free-choice trials, two go-stimuli were presented, and participants were instructed that in these cases, they were free to choose which task to perform. In forced-no-go trials, one go- and one no-go stimulus were presented, and participants were instructed that in these cases, they should perform the task requiring a response. Critically, the two stimuli in each of these trials were separated by a random short (50 ms) or long (300 ms) SOA. In this experiment, we additionally included some forced-single trials where only one stimulus was presented. It was always a go stimulus, and participants were instructed that in these cases, they had to perform the task associated with the presented stimulus. Performance in these trials can be compared with that in forced-no-go trials to determine whether no-go stimuli are processed in parallel. Specifically, we will compare S_1_-go in forced-no-go trials with S_1_-go in forced-single trials. Resource-sharing models suggest that S_1_-go will be slowed by the presence of an S_2_-no-go stimulus if capacity-limited S_2_-no-go processing withdraws some resources so that it can be processed in parallel with the S_1_-go stimulus. On the other hand, bottleneck models suggest that an S_2_-no-go stimulus would simply be held waiting for bottleneck access and thus should have no effect on the RT to the S_1_-go stimulus.

### Method

#### Participants

40 participants were tested online, but the data of 6 participants had to be excluded (cf. preregistration and data preparation section). Thus, the final sample consisted of 34 people (22 women, 28 right-handed), who ranged in age from 18 to 55 years (M = 22.47).

#### Sample size justification

The sample sizes in the two experiments were somewhat arbitrarily set, but both practical constraints (e.g., participant availability) and empirical constraints (e.g., effect size in previous studies) were taken into account. For example, we ensured that our power was sufficient to detect potential effects of both switch costs (Mittelstädt et al., 2019, $\eta ^2_p$. = .82, cf. Experiment 1a in) and PRP-like effects in no-go trials (Mittelstädt & Miller, 2017, $\eta ^2_p$ = .27, cf. Experiment 2 in), with a power level of 80% and a significance level of 5%. This power analysis, based on the smaller effect size observed in Mittelstädt and Miller (2017), would have suggested 24 participants. However, we aimed for a larger sample size as we anticipated it would be necessary to exclude some participants, and also because we wanted to see whether other potential effects (e.g., costs when switching between processing modes) would be found in this new paradigm. Furthermore, for both experiments reported in the main text, the results were very similar when including the participants that were excluded based on strong global task choice strategies (leading to N = 37 and N = 38 in Experiment 1 and Experiment 2, respectively). Finally, these result patterns were also similar when combining the data of both experiments (omitting the forced-single trials of Experiment 1).

#### Apparatus and stimuli

The experiment was conducted online using the JavaScript library jsPsych (De Leeuw, 2015). All visual stimuli were presented in black on a grey background. A centrally positioned plus sign served as the fixation point. The stimuli were the digits 1–9 for the number task and the uppercase letters A, B, C, L, M, N, X, Y, and Z for the letter task. For the number task, participants had to press a left versus right key when the number was smaller than 4 versus larger than 6 and to not respond when the number was in the range from 4 to 6 (i.e., 4, 5, or 6). For the letter task, participants had to press a left versus right key when the letter was before L versus after N in the alphabet and to not respond when the letter was in the range from L to N (i.e., L, M, or N). Thus, we used the same tasks as Fröber and Dreisbach (2017) except that no-go stimuli were also part of the stimulus set (i.e., hereafter “no-go-stimuli”, whereas the other stimuli are labelled as “go-stimuli”). The stimuli of the two tasks appeared one above the other at the center of the screen. The task-specific stimulus positions were kept constant throughout the experiment, but counterbalanced across participants. For half of the participants, the letter appeared at the top, whereas for the other half of participants the positions were reversed. Responses to the task stimuli at the top were always made by key presses with the index and middle fingers of the left hand (“Q” and “W”), whereas responses to the task stimuli at the bottom were always made by key presses with the index and middle fingers of the right hand (“O” and “P”).

#### Procedure

Participants were tested in 8 blocks of 96 trials per block (i.e., 768 trials in total). The first two blocks were considered practice and were not included in the analysis. As can be seen in the trial table (Table 1), each experimental block consisted of 50% free-choice (48 trials) and 50% forced-choice (48 trials) randomly intermixed trials. The specific combinations were equally distributed across the free-choice trials (i.e., stimulus order [letter: S_1_ & number: S_2_ vs. number: S_1_ & letter: S_2_] X SOA [50 ms vs. 300 ms]). Note that there were two types of forced-choice trials—that is, trials in which only one stimulus appeared and trials in which the other stimulus was a no-go stimulus. Thus, for forced-choice trials with no-go stimuli there were the following combinations: Stimulus order [letter: S_1_ & number: S_2_ vs. number: S_1_ & letter: S_2_] $\times$ forced task [letter vs. number] $\times$ SOA [50 ms vs. 300 ms]. For forced-choice type trials with only one stimulus, there were only the two conditions of whether the presented stimulus (forced task) was a letter or number.

**Table 1:** Overview of the different trial types in Experiment 1

N per Block	Mode	SOA	Stimulus order
24	Free	50	S_1_-go & S_2_-go
24	Free	300	S_1_-go & S_2_-go
8	Forced-no-go	50	S_1_-go & S_2_-no-go
8	Forced-no-go	50	S_1_-no-go & S_2_-go
8	Forced-no-go	300	S_1_-go & S_2_-no-go
8	Forced-no-go	300	S_1_-no-go & S_2_-go
16	Forced-single	NA	S_1_-go

See text for more details. Within each trial type, the letter and number stimuli each appeared first in half of the trials. Within each of the forced-no-go and forced-single trial types, there were equal numbers of trials requiring responses to the letter and number tasks for each stimulus order

Participants were instructed that in free-choice trials (i.e., when both stimuli required a response), they were free to perform whichever task they wanted. However, in forced-choice trials (i.e., when only one stimulus appeared or only one stimulus required a response) they had to perform the task related to the go-stimulus (see Fig. 1).

At the beginning of each trial, the fixation cross appeared on the screen for 500 ms. In free-choice trials, S_1_ (letter or number) was displayed immediately at the offset of the fixation cross and S_2_ was added to the display at the end of that trial’s SOA, with both S_1_ and S_2_ being go-stimuli. In forced-choice trials, S_1_ was also displayed at the offset of the fixation cross, but S_2_ was only added to the display in forced-choice trials with no-go stimuli (i.e., S_2_ was a go stimulus if S_1_ was a no-go stimulus and vice versa). The specific identities of stimuli in a given trial were selected randomly with the constraint that none of the stimuli had been presented in the previous trial. The stimulus (stimuli) remained on the screen until the participant responded, or up to a response deadline of 4 s. Following correct responses, a screen with the fixation cross with an intertrial interval (ITI) of 500 ms was presented before the next trial started. In case of an error (or no response within the response deadline), an additional error screen was presented for 3 s (first practice block: 4 s) indicating that an error had been made (or that the response was too slow) and repeating the S-R mappings for the two tasks. RTs were measured from the onset of the stimulus related to the task that the participant performed until the key press.

#### Data preparation

The first two blocks and the first trial of each block were excluded from all analyses.^1^ We also excluded trials with RTs less than 200 ms (including trials in which a response was given prior to onset of the stimulus related to the performed task, 0.5%), trials without any response within the RT measurement interval (1.9%), and post-error trials (6.0%). Furthermore, we additionally excluded trials in which participants responded to the wrong task (4.3%) in forced-choice trials. Note that the patterns were similar when including these trials as errors in the PE analyses. For task choice (and RT) analyses, we additionally excluded any erroneous trials.

After our data trimming procedure, we examined whether participants followed any consistent global task choice strategies in free-choice trials (i.e., always selecting the same task or always selecting the same or different task that was performed in the previous trial) which may overshadow any potential effects of the central manipulations. We excluded the data of five participants who selected one of the two tasks in >95% of trials and/or switched or repeated tasks in >95%. Finally, we excluded the data of one additional participant due to exceptionally long mean reaction times (mean RT of 1928 ms after the data trimming procedure). Note that excluding data of participants based on overall task performance was only preregistered in Experiment 2 but not Experiment 1. Although the results were very similar without excluding the data of this participant in Experiment 1, we decided to apply the same data preparation procedure across both experiments.

### Results

For this and the next experiment, we initially performed all subsequent analyses distinguishing between tasks (i.e., letter and number). Since none of the analyses revealed any effects or interactions involving this factor, we collapsed across tasks for all reported analyses. In the following, we first present the forced-choice task performance results (i.e., RT and PE) on trial N, without considering any previous trial characteristics. Subsequently, we report these results while taking into account whether the processing mode was free or forced and which task participants performed in the previous trial. Similarly, we first report the impact of SOA on task choice behavior, measured by the percentage of selecting the task associated with the first presented stimulus. Then, we further analyze this data by considering the previous trial’s mode and task. Note that we only mentioned previous trial characteristics in our preregistration for Experiment 2, not in Experiment 1. For completeness, we include the results of free-choice task performance (i.e., RT and PE) in "Appendix A".

#### Dynamics—forced: trial N alone

**Fig. 2:**
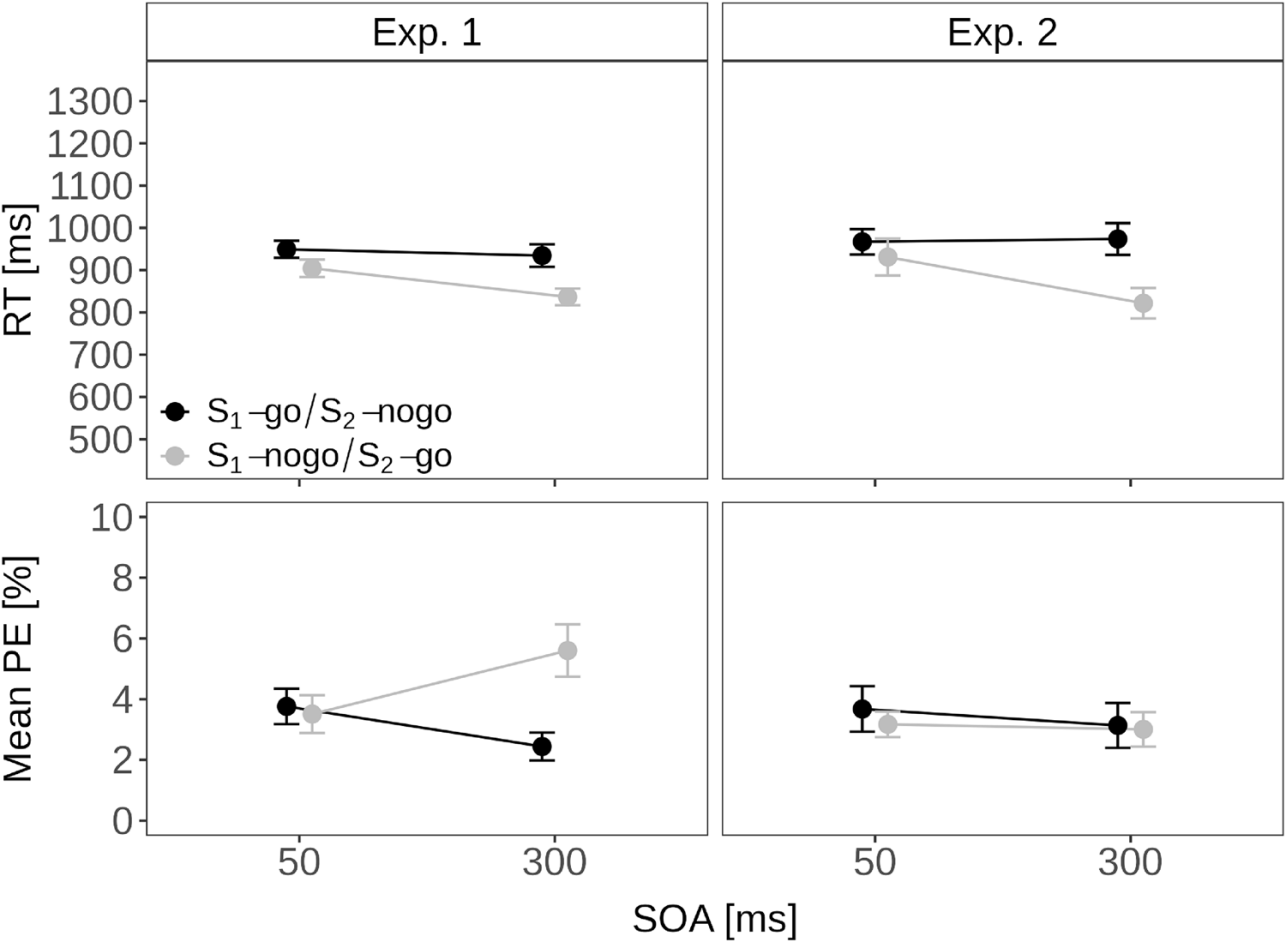
Dynamics—forced: Trial N alone as a function of stimulus order and SOA in Experiment 1 and Experiment 2. Note. Mean reaction time (RT; top-row) and mean percentage error (PE; bottom-row) in Experiment 1 (left column) and Experiment 2 (right column). For comparison, the mean RT and PE for forced-single trials in Experiment 1 were 823 ms and 3.5%

Figure 2 shows the mean RT and mean PE for forced-choice trials in trials with two stimuli (i.e., one no-go and one go-stimulus) as a function of stimulus order (S_1_-no-go and S_2_-go vs. S_1_-go and S_2_-no-go) and SOA (short vs. long).

An ANOVA with these two factors on mean RT revealed that all effects were significant: The main effect of stimulus order indicated faster responses for S_1_-no-go than S_1_-go trials (870 vs. 942 ms), *F*(1, 33) = 33.34, *p* < 0.001, $\eta ^2_p$ = 0.50. The main effect of SOA indicated faster responses at long compared to short SOA (886 vs. 927 ms), *F*(1, 33) = 9.98, *p* = 0.004, $\eta ^2_p$ = 0.23. The interaction indicated that the RT advantage at long over short SOA was larger when S_1_ was the no-go stimulus (837 vs. 904 ms) than when S_1_ was the go stimulus (934 vs. 949 ms), *F*(1, 33) = 4.60, *p* = 0.039, $\eta ^2_p$ = 0.12. Thus, in particular, the PRP-like SOA effect when responding to an S_2_-go stimulus supports the idea that participants have also processed the no-go S_1_ (cf. Mittelstädt & Miller, 2017, Miller & Durst, 2015). In other words, this effect suggests that making a no-go decision also requires resource-limited selection processes.

A parallel ANOVA on mean PE in the two-stimulus trials revealed a significant main effect of stimulus order reflecting larger mean PE for S_1_-no-go than S_1_-go trials (4.6% vs. 3.1%), *F*(1, 33) = 8.23, *p* = 0.007, $\eta ^2_p$ = 0.20. The interaction was also significant indicating fewer errors at long compared to short SOA when S_1_ was the go stimulus (2.4 vs. 3.8%), but more errors at long compared to short SOA when S_1_ was the no-go stimulus (5.6 vs. 3.5%), *F*(1, 33) = 7.97, *p* = 0.008, $\eta ^2_p$ = 0.19. Thus, the mean PE pattern warrants some caution when interpreting the RT results due to the presence of a speed-accuracy tradeoff.

As a further check that the no-go stimuli were processed, we compared performance in the forced-no-go trials where S_1_ was the go-stimulus with performance in the forced-single trials (where S_1_ was always the go-stimulus). Thus, these analyses allowed us to investigate the influence of the presence or absence of a no-go S_2_ on S_1_ processing. If S_1_-go processing is slower with the onset of an S_2_-no-go, it would indicate that the S_2_-no-go withdraws some resources to be processed in parallel with S_1_-go (see, Mittelstädt & Miller, 2017, for a similar argument). The ANOVA on the corresponding mean RTs with the within-subject factor of forced-choice type (i.e., no-go stimulus after short SOA, no-go stimulus after long SOA, no no-go stimulus) revealed a significant effect, *F*(2, 66) = 32.72, *p* < 0.001, $\eta ^2_p$ = 0.50, indicating that forced-single trials (823 ms) were substantially faster than forced-no-go at both short (949 ms) and long (934 ms) SOAs (with *p* < 0.001 for both pairwise comparisons). The ANOVA on the corresponding mean PEs revealed no significant effect, *F*(2, 66) = 2.44, *p* = 0.095, $\eta ^2_p$ = 0.07. Descriptively, forced-single trials (3.5%) had smaller PEs than forced-no-go trials at short SOA (4.3%), but larger PEs than forced-no-go trials at long SOA (3.0%). Thus, the forced-single versus forced-no-go at short SOA comparison was not complicated by a possible SAT.

#### Dynamics—forced: sequential effects

**Fig. 3:**
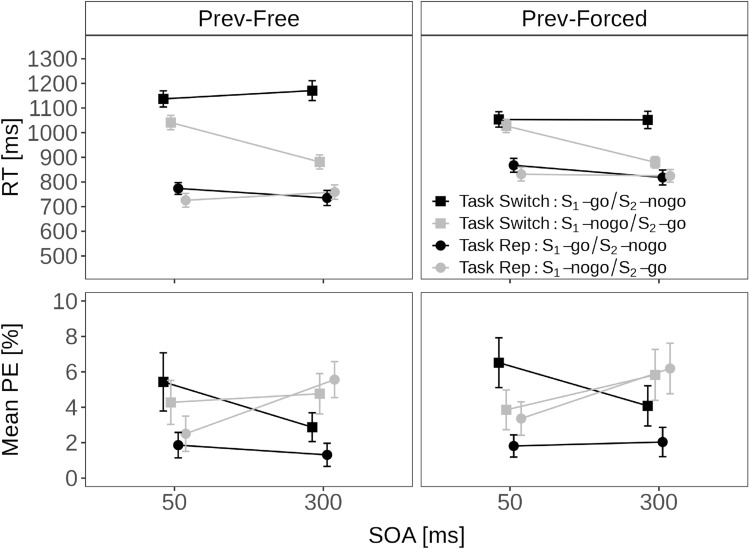
Dynamics—forced: Sequential Effects in Experiment 1. Note. Mean reaction time (RT: top-row) and mean percentage error (PE; bottom-row) for forced-choice trials with two stimuli in a current trial as a function of stimulus order (S_1_-no-go and S_2_-go vs. S_1_-go and S_2_-no-go) and task transition (repetition vs. switch) separately for trials preceded by free-choice trials (left column) and forced-choice trials (right column)

Figure 3 shows the mean RT and mean PE for forced-choice trials with two stimuli (i.e., one no-go and one go-stimulus) as a function of stimulus order (S_1_-no-go and S_2_-go vs. S_1_-go and S_2_-no-go), SOA (short vs. long), previous mode (free, forced), and task transition (repetition, switch).

An ANOVA with these factors on mean RT revealed that the main effect of task transition was significant, indicating smaller RTs for repetition than switch trials (792 vs. 1030 ms), *F*(1, 33) = 167.78, *p* < 0.001, $\eta ^2_p$ = 0.84. The main effect of stimulus order was also significant, indicating smaller RTs for S_1_-no-go than S_1_-go trials (871 vs. 951 ms), *F*(1, 33) = 35.10, *p* < 0.001, $\eta ^2_p$ = 0.52. The main effect of SOA was also significant, indicating smaller RTs at long than short SOA (890 vs. 932 ms), *F*(1, 33) = 11.21, *p* = 0.002, $\eta ^2_p$ = 0.25. The 2-way interaction between task transition and stimulus order was significant, *F*(1, 33) = 54.41, *p* < 0.001, $\eta ^2_p$ = 0.62. Switch costs were larger when S_1_ was the go-stimulus (799 vs. 1103 ms) than when S_2_ was the go-stimulus (785 vs. 957 ms). The 2-way interaction between task transition and SOA was significant, *F*(1, 33) = 5.37, *p* = 0.027, $\eta ^2_p$ = 0.14. This interaction indicates larger switch costs at the short (800 vs. 1065 ms) than long SOA (784 vs. 996 ms). There was also a 2-way interaction between stimulus order and SOA, *F*(1, 33) = 5.95, *p* = 0.020, $\eta ^2_p$ = 0.15. This interaction indicated that RTs were only slightly larger at short compared to long SOA when S_1_ was the go-stimulus (958 vs. 944 ms), but this SOA-based RT advantage was larger for S_1_-no-go trials (906 vs. 836 ms). In other words, when participants were required to respond to the second stimulus (i.e., S_2_-go), RT significantly decreased as the SOA increased. As mentioned above, this finding mirrors the decrease in second-task RT with SOA observed in dual-task studies, suggesting that the processing of both S_1_-no-go and S_1_-go demands limited cognitive resources, which in turn, leads to a substantial PRP-like effect in these trials. There was also a 2-way interaction between task transition and previous mode, *F*(1, 33) = 21.75, *p* < 0.001, $\eta ^2_p$ = 0.40. This interaction indicated that switch costs were generally larger when the previous response was free (748 vs. 1058 ms) compared to forced (836 vs. 1003 ms). Moreover, there was a 2-way interaction between stimulus order and previous mode, *F*(1, 33) = 5.55, *p* = 0.025, $\eta ^2_p$ = 0.14. Responses were generally faster in S_1_-no-go than S_1_-go trials, but this difference was larger when the previous response was free (851 vs. 954 ms) than forced (891 vs. 948 ms). There was also a 3-way interaction between task transition, stimulus order, and SOA, *F*(1, 33) = 29.94, *p* < .001, $\eta ^2_p$ = .48. This interaction indicated that the PRP-like effect measured when participants responded to S_2_-go was larger when a switch was required compared to repetition. Separate ANOVAs for each task transition condition revealed that the 2-way interactions between stimulus order and SOA were (marginal) significant when a switch (*p* = .007, $\eta ^2_p$ = 0.21) and a repetition was required (*p* = 0.053, $\eta ^2_p$ = 0.11). Moreover, responses to S_1_-go were only faster at long compared to short SOA when a repetition compared to switch was required, suggesting that switch-S_2_-no-go interfered more strongly with S_1_-go processing than repetition–S_2_-go. Finally, there was a 3-way interaction between task transition, stimulus order, and previous mode, *F*(1, 33) = 5.23, *p* = 0.029, $\eta ^2_p$ = 0.14. Switch costs were larger when the previous response was free than forced when S_1_ was the go-stimulus, but this difference was reduced when S_2_ was the go-stimulus. In separate ANOVAs for each stimulus order, switch costs were larger when the previous response was free than forced both when S_1_ was the go-stimulus (*p* < 0.001, $\eta ^2_p$ = 0.38) and when S_2_ was the go-stimulus (*p* = 0.005, $\eta ^2_p$ = 0.22).

A parallel ANOVA on mean PEs revealed a significant main effect of task transition reflecting switch costs (3.1% vs. 4.7%), *F*(1, 33) = 7.97, *p* = 0.008, $\eta ^2_p$ = 0.19. The main effect of stimulus order was also significant indicating larger PEs for S_1_-no-go than S_1_-go trials (4.5% vs. 3.2%), *F*(1, 33) = 5.59, *p* = 0.019, $\eta ^2_p$ = 0.14. There was also a 2-way interaction between stimulus order and SOA, *F*(1, 33) = 7.42, *p* = 0.010, $\eta ^2_p$ = 0.18. This interaction reflected the fact that PE were larger at short compared to long SOA when S_1_ was the go-stimulus (3.9% vs. 2.6%), but PEs were smaller at short compared to long SOA when S_1_ was the no-go stimulus (3.5% vs. 5.8%). Moreover, the 2-way interaction between stimulus order and task transition was significant, *F*(1, 33) = 6.52, *p* = 0.015, $\eta ^2_p$ = 0.15. Switch costs were larger for S_1_-go (1.8% vs. 4.7%) than for S_2_-go trials (4.4% vs. 4.7%). Finally, there was a significant 2-way interaction between SOA and task transition, *F*(1, 33) = 6.81, *p* = 0.013, $\eta ^2_p$ = 0.17. This interaction indicates larger switch costs at the short SOA (2.4% vs. 5.0%) than at the long SOA (3.8% vs. 4.4%).

#### Task choice—free: trial N alone

Overall, there was a strong preference to select S_1_ over S_2_ (62.9%) as indicated by a significant *t*-test against chance, *t*(33) = 11.19, *p* < 0.001, *d* = 1.95. A paired *t*-test indicated that the percentage of S_1_-task choices was larger at the long (73.7%) compared to short (52.2%) SOA, *t*(33) = 10.76, *p* < 0.001, *d* = 1.85.

#### Task choice—free: sequential effects

**Fig. 4:**
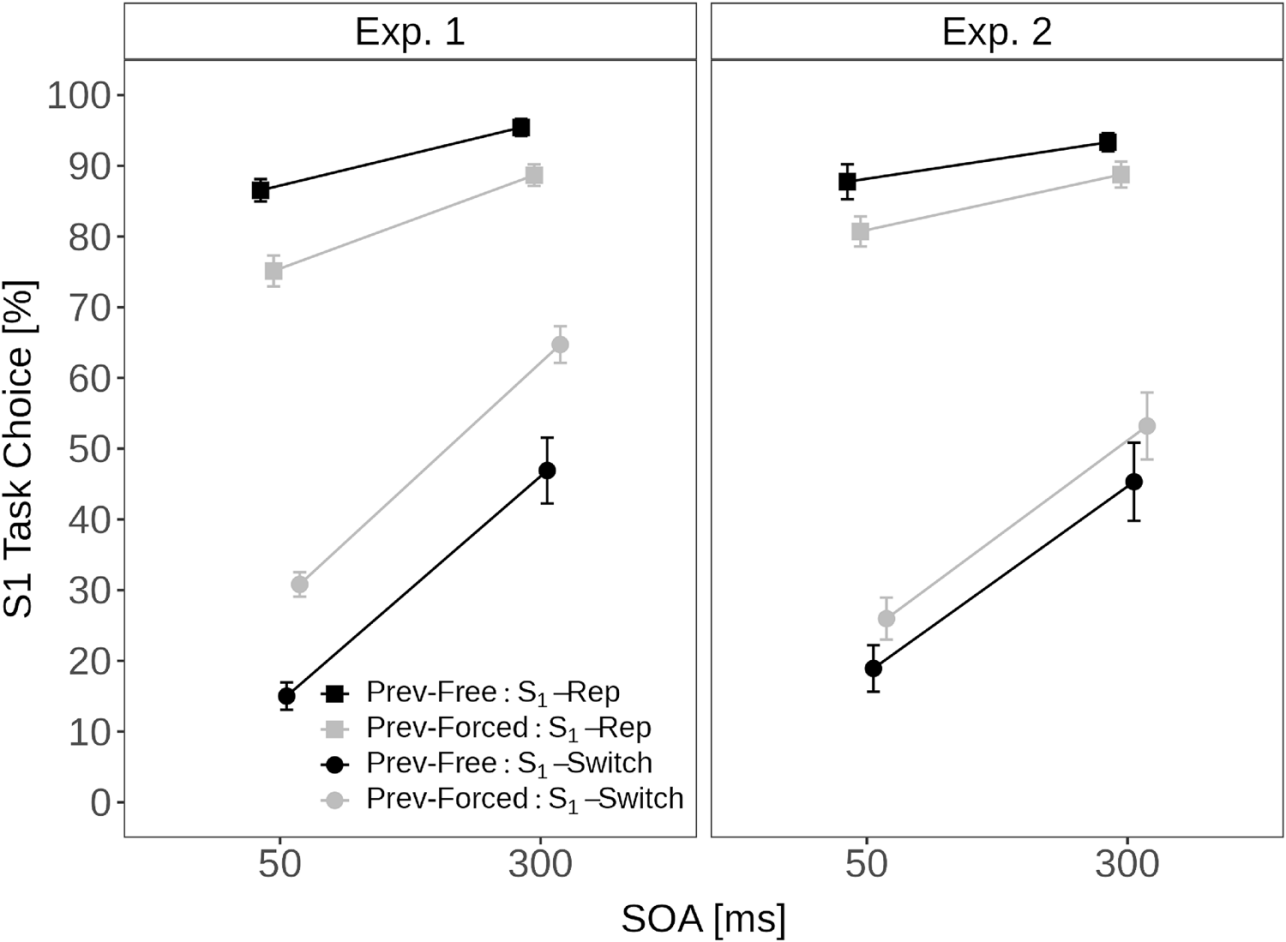
Task choice—free: sequential effects. Note. Percentage of S_1_ choices in free-choice trials as a function of SOA (short, long), previous trial mode (free vs. forced) and S_1_ transition type (repetition-S_1_ vs. switch-S_1_) separately for Experiments 1 (left column) and 2 (right column)

Figure 4 shows the S_1_-task percentages as a function of SOA and previous response mode separately for whether S_1_ was associated with switching vs. repeating tasks.

The ANOVA revealed that all main effects were significant: the main effect of SOA indicated a stronger preference for S_1_ with long (73.9%) compared to short SOA (51.9%), *F*(1, 33) = 142.78, *p* < 0.001, $\eta ^2_p$ = 0.81. The main effect of S_1_ transition type reflected a task repetition bias—that is, participants had a stronger preference for S_1_ when this stimulus was associated with repeating (86.4%) as compared to switching tasks (39.4%), *F*(1, 33) = 347.82, *p* < 0.001, $\eta ^2_p$ = 0.91. The main effect of previous mode indicated that participants’ preference for S_1_ was reduced when the previous trial was free (61.0%) as compared to forced (64.8%), *F*(1, 33) = 18.53, *p* < 0.001, $\eta ^2_p$ = 0.36. There was also a significant 2-way interaction between S_1_ transition type and previous response mode, *F*(1, 33) = 43.06, *p* < 0.001, $\eta ^2_p$ = 0.57. Participants’ preference for S_1_-repetitions over S_1_-switches was stronger when the previous mode was free (91.0% vs. 31.0%) than when the previous mode was forced (81.9% vs. 47.8%). In other words, participants were more likely to repeat a freely-chosen task than a forced-chosen task. Furthermore, there was a significant 2-way interaction between SOA and S_1_ transition type, *F*(1, 33) = 76.83, *p* < 0.001, $\eta ^2_p$ = 0.70. The preference to select S_1_ at short compared to long SOA was larger when S_1_ was associated with switching (22.9% vs. 55.8%) as compared to repeating tasks (80.8% vs. 92.0%). While this interaction may suggest that participants particularly rely on external factors when deciding to switch tasks, this interaction may solely arise for statistical reasons—that is, the bias to select the task associated with S_1_ was already at ceiling when S_1_ was a repetition stimulus. The two-way interaction between previous mode and SOA (*p* = 0.087, $\eta ^2_p$ = 0.09) and the three-way interaction (*p* = 0.447, $\eta ^2_p$ = 0.02) were not significant.

### Discussion

The results of Experiment 1 replicate and extend a variety of results from task-switching and dual-tasking paradigms into a new multitasking choice/no-go paradigm—free of motor interference—in which participants can freely choose which task to process initially in every trial, unconstrained by experimenter-determined requirements to process one specific task or to process the two tasks in a specific order.

First, as in task-switching paradigms, performance was clearly better in task repetition trials than in task switch trials, replicating the task-switching decrement found when the to-be-performed task is cued by the experimenter (e.g., Dreisbach & Haider, 2006, Koch & Allport, 2006, Gade & Koch, 2007). Moreover, voluntary task choices indicated that participants clearly preferred task repetitions to task switches, presumably because of the smaller cognitive effort required for repetitions (e.g., Mendl & Dreisbach, 2022). Both the task-switching decrement and the task repetition bias were modulated by SOA, showing that participants’ dual-tasking behavior was sensitive to the environmental demands on the limited-capacity cognitive processes responsible for switch costs. Finally, in addition to the time-consuming processes involved in task switching, participants were also slower when performing the same tasks but switching from a free to a forced processing mode (or from a forced to a free processing mode, see "Appendix A") (e.g., Qiao et al., 2023). This suggests that free and forced tasks are at least partially differentially internally represented-a finding that aligns well with the observation that participants were also more strongly biased to repeat free-choice tasks than forced-choice tasks.

Second, a PRP-like effect of SOA was evident when participants had to respond to S_2_ because S_1_ was a no-go stimulus: RT decreased dramatically as SOA increased, which is typically regarded as a sign that S_1_ processing delays the response to S_2_ at short SOAs (e.g., Ruthruff et al., 2001, Pashler, 1994, Miller & Durst, 2015). Furthermore, based on the slowing of RTs to S_1_ when S_2_ is present than when it is absent, these limitations seem to be better explained by the allocation of limited cognitive resources for parallel task processing, rather than the presence of a structural response selection bottleneck (cf. Mittelstädt & Miller, 2017). Interestingly, the PRP-like effect on RTs to S_2_ was stronger for switch compared to repetition trials—a comparison that is not available in standard dual-tasking paradigms where all trials are switch trials. Following up on resource-sharing accounts, this difference may suggest that the degree of engagement in parallel processing of S_2_ seems to be limited by the additional cognitive demands that must be overcome when switching tasks (e.g., task-set reconfiguration). Bottleneck models would naturally make the opposite prediction (i.e., smaller SOA effect for S_2_ switch than repetition trials): When S_2_ is a switch stimulus, S_1_ is a repetition stimulus, which may require less time to categorize as a no-go stimulus, leading to a reduced SOA effect. With additional assumptions, bottleneck models may be extended to account for the opposite pattern. However, since other patterns in this experiment are also better explained by resource models, it seems reasonable to prefer the more straightforward explanation of resource models here as well.

## Experiment 2

The purpose of Experiment 2 was to investigate which effects would replicate when omitting the S_1_-only trials that were included in Experiment 1. For example, in Experiment 1, performance at the long SOA might have been affected by uncertainty about whether S_2_ would be presented at all, since we did include some S_1_-only trials. Thus, participants might have briefly thought that S_1_ was going to be the only option on that trial, and this may have biased them to increasingly select S_1_ at long compared to short SOAs, which we would not normally see when using a pure version of the choice-no-go paradigm without single-stimulus trials. Moreover, in the sequential analyses, we collapsed across previous-forced-single and previous-forced-no-go trials when comparing previous-free versus previous-forced, as the number of trials per condition did not allow considering another distinction (e.g., many participants would have no or only a few trials in some of the cells of the forced-choice dynamic analyses). Thus, by using only forced-no-go and forced-free trials, we can also directly investigate the impact of a previous-forced-no-go trial compared to a previous-free-choice trial.

### Method

We again tested 40 people online, but we excluded the data of 12 participants who selected one of the two tasks in >95% of trials and/or switched or repeated tasks in >95%. Moreover, one additional participant was excluded due to accuracy below 80%. The final 27 participants (23 right-handed, 17 female) ranged in age from 18 to 32 years (M = 22.9). The apparatus, stimuli, and procedure were the same as in Experiment 1 except that we replaced the S_1_-only forced-choice trials with forced-choice trials with no-go stimuli. Thus, two stimuli were always presented in both forced-no-go and free-choice trials.

#### Data preparation

We followed the same data preparation procedure as in Experiment 1. Thus, trials with RTs less than 200 ms (0.1%), trials without any response within the RT interval (2.8%), post-error trials (4.8%), and trials in which participants did choose the wrong task (5.5%) were excluded from all analyses.

### Results

We conducted the same basic data analyses, and hence the results are presented in a comparable manner to Experiment 1, with the corresponding free-choice task performance results in "Appendix B". However, it is important to emphasize that in this experiment there were no forced-single trials, as we only used forced-no-go trials in addition to the free-choice trials.

#### Dynamics—forced: trial N alone

Figure 2 shows the mean RT and mean PE for forced-choice trials with two stimuli (i.e., one no-go and one go-stimulus) as a function of stimulus order (S_1_-no-go and S_2_-go vs. S_1_-go and S_2_-no-go) and SOA (short vs. long).

An ANOVA with these two factors on mean RT revealed again that all effects were significant: The main effect of stimulus order indicated faster responses for S_1_-no-go than S_1_-go trials (876 vs. 970 ms), *F*(1, 26) = 18.83, *p* < 0.001, $\eta ^2_p$ = 0.42. The main effect of SOA indicated faster responses at long compared to short SOA (898 vs. 949 ms), *F*(1, 26) = 14.95, *p* = 0.001, $\eta ^2_p$ = 0.37. The interaction indicated that RTs were substantially slower at short over long SOA when S_1_ was the no-go stimulus (931 vs. 821 ms), but, if anything, slightly faster at short than long SOA when S_1_ was the go stimulus (967 vs. 974 ms), *F*(1, 26) = 23.84, *p* < 0.001, $\eta ^2_p$ = 0.48.

In contrast to Experiment 1, there were no signs of any speed-accuracy tradeoffs (see Fig. 2) and the ANOVA on PE revealed no significant effects (all *p* > 0.398, all $\eta ^2_p$ < 0.04).

#### Dynamics—forced: sequential effects

**Fig. 5:**
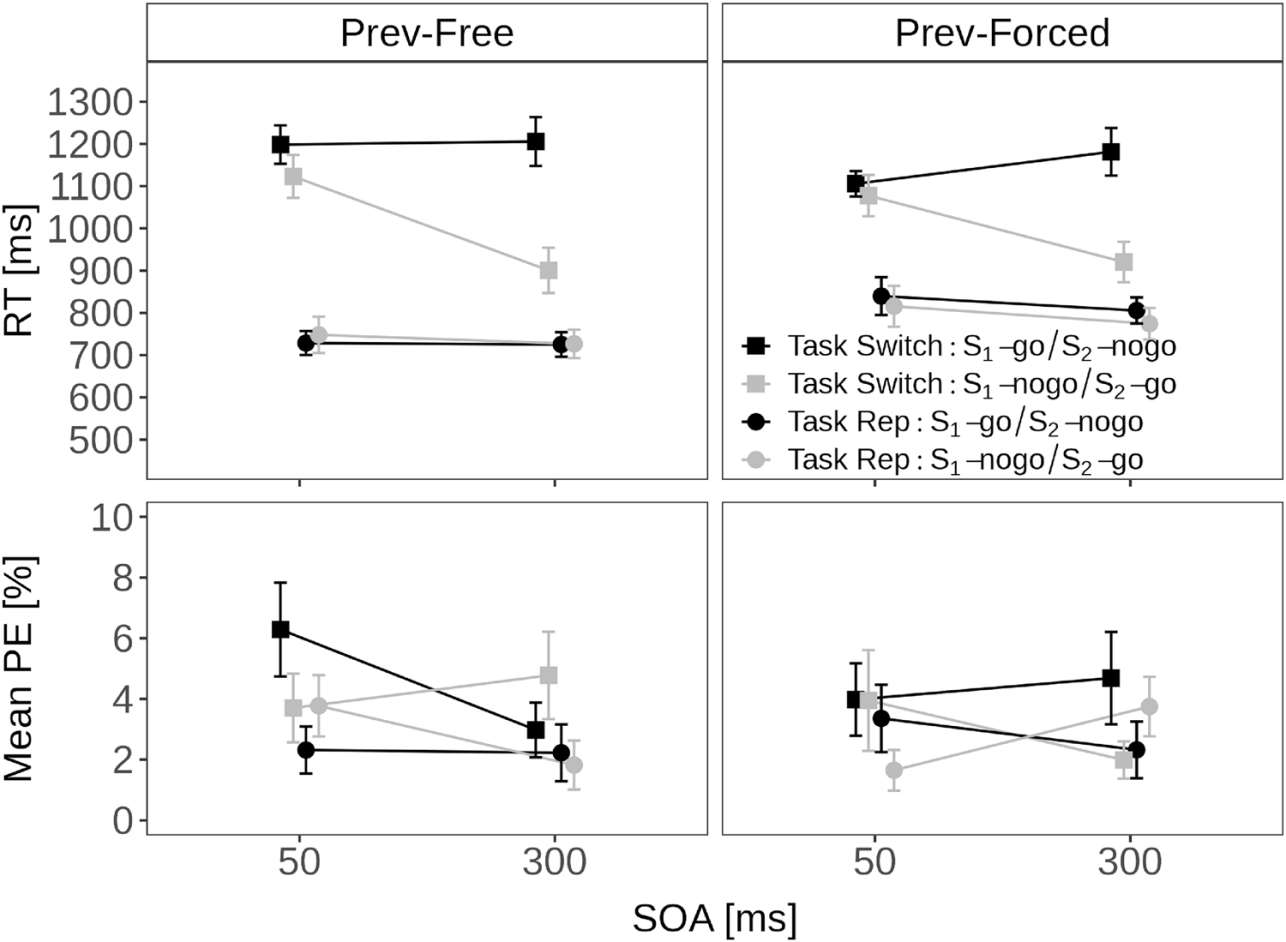
Dynamics—forced: sequential effects in Experiment 2. Note. Mean reaction time (RT: top-row) and mean percentage error (PE; bottom-row) for forced-choice trials with two stimuli in a current trial as a function of stimulus order (S_1_-no-go and S_2_-go vs. S_1_-go and S_2_-no-go) and task transition (repetition vs. switch) separately for trials preceded by free-choice trials (left column) and forced-choice trials (right column)

Figure 5 shows the mean RT and mean PE for forced-choice trials with two stimuli (i.e., one no-go and one go-stimulus) as a function of stimulus order (S_1_-no-go and S_2_-go vs. S_1_-go and S_2_-no-go), SOA (short vs. long), previous mode (free, forced), and task transition (task repetition, task switch).

An ANOVA with these four factors on mean RTs revealed a significant main effect of transition, with smaller RTs for task repetitions than task switches (770 vs. 1089 ms), *F*(1, 26) = 105.26, *p* < 0.001, $\eta ^2_p$ = .80. The main effect of stimulus order was also significant, indicating smaller RTs for S_1_-no-go than S_1_-go trials (886 vs. 978 ms), *F*(1, 26) = 13.20, *p* < 0.001, $\eta ^2_p$ = 0.34. The main effect of SOA was also significant, indicating smaller RTs at long than short SOA (905 vs. 955 ms), *F*(1, 26) = 12.81, *p* = 0.001, $\eta ^2_p$ = 0.34. There was also a significant 2-way interaction between stimulus order and SOA, *F*(1, 26) = 28.61, *p* < 0.001, $\eta ^2_p$ = 0.52. As in Experiment 1, this interaction reflected the fact that RTs were considerably larger at short compared to long SOA when S_1_ was the no-go-stimulus (941 vs. 830 ms), whereas RTs were slightly smaller at short compared to long SOA when S_1_ was the go-stimulus (968 vs. 979 ms). The 2-way interaction between stimulus order and task transition was also significant, *F*(1, 26) = 52.54, *p* < 0.001, $\eta ^2_p$ = 0.67. Switch costs were larger when S_1_ was the go-stimulus (775 vs. 1172 ms) than when S_1_ was the no-go stimulus (766 vs. 1005 ms). There was also a 2-way interaction between task transition and previous mode, *F*(1, 26) = 15.21, *p* < 0.001, $\eta ^2_p$ = 0.37. This interaction indicated that switch costs were generally larger when the previous response was free (732 vs. 1107 ms) compared to forced (809 vs. 1071 ms). There was also a 2-way interaction between SOA and task transition, *F*(1, 26) = 6.75, *p* = 0.015, $\eta ^2_p$ = 0.21. This interaction indicates smaller switch costs at long (758 vs. 1052 ms) compared to short SOA (783 vs. 1126 ms). The 3-way interaction between task transition, stimulus order, and SOA was again significant, *F*(1, 26) = 25.48, *p* < 0.001, $\eta ^2_p$ = 0.49. As in Experiment 1, the PRP-like effect was larger when participants processed a switch-S_2_-go than a repetition-S_2_-go, and responses to repetition-S_1_-go were particularly slowed down at short compared to long SOA. Separate ANOVAs for each task transition condition revealed that the 2-way interactions between stimulus order and SOA were significant when a switch (*p* < 0.001, $\eta ^2_p$ = 0.48) but not when a repetition was required (*p* = 0.225, $\eta ^2_p$ = 0.06). The 3-way interaction between task transition, stimulus order, and previous mode was marginally significant, *F*(1, 26) = 4.12, *p* = 0.053, $\eta ^2_p$ = 0.14. As in Experiment 1, switch costs were significantly larger when the previous response was free than it was forced both when S_1_ was the go-stimulus (*p* < 0.001, $\eta ^2_p$ = 0.40) and when S_2_ was the go-stimulus (*p* = 0.048, $\eta ^2_p$ = 0.14), but the stronger interaction for go S_1_’s produced the marginally significant 3-way interaction. Finally, the 3-way interaction between task transition, SOA, and previous mode was significant, *F*(1, 26) = 7.95, *p* = 0.009, $\eta ^2_p$ = 0.23. There were smaller switch costs at long compared to short SOA when the previous mode was free, but slightly larger switch costs at long compared to short SOA when the previous response was forced. Separate ANOVAs for each previous mode condition revealed that the 2-way interaction between SOA and task transition was only significant when the previous mode was free (*p* < 0.001, $\eta ^2_p$ = 0.49) but not when it was forced (*p* < 0.918, $\eta ^2_p$ < 0.01).

A parallel ANOVA on mean PEs revealed fewer errors to repetition compared to switch trials (2.7% vs. 4.0%), *F*(1, 26) = 6.85, *p* = 0.015, $\eta ^2_p$ = 0.21. There was also a significant 4-way interaction between all factors, *F*(1, 26) = 9.60, *p* = 0.005, $\eta ^2_p$ = 0.27. When the previous response was forced, there were larger switch costs at long compared to short SOA for S_1_-go/S_2_-no-go, whereas switch costs were smaller (and even reversed) at long compared to short SOA for S_1_-no-go/S_2_-go. When the previous response was forced, there were larger switch costs at long compared to short SOA for S_1_-go/S_2_-no-go, whereas switch costs were smaller (and even reversed) at long compared to short SOA for S_1_-no-go/S_2_-go.

#### Task choice—free: trial N alone

Overall, there was again a strong preference to select S_1_ over S_2_ (61.7%) as indicated by a significant *t*-test against chance, *t*(26) = 6.36, *p* < 0.001, *d* = 1.2. A paired *t*-test indicated that S_1_-task choices were more common at long (69.8%) compared to short (53.4%) SOA, *t*(26) = 6.30, *p* < 0.001, *d* = 1.2.

#### Task choice—free: sequential effects

Figure 4 shows the S_1_-task percentages as a function of SOA and previous response mode separately for whether S_1_ was associated with switching versus repeating tasks. The ANOVA revealed a significant main effect of SOA, with a stronger preference for S_1_ with long (70.1%) compared to short SOA (53.3%), *F*(1, 26) = 50.16, *p* < 0.001, $\eta ^2_p$ = 0.66. The significant main effect of S_1_ transition type indicated that participants were more likely to select S_1_ for repeating (87.6%) than for switching tasks (35.9%), *F*(1, 26) = 182.03, *p* < 0.001, $\eta ^2_p$ = 0.88. Contrary to Experiment 1, the main effect of previous mode was not significant (*p* = 0.379, $\eta ^2_p$ = 0.03). However, as in Experiment 1, there was a significant 2-way interaction between S_1_ transition type and previous response mode, *F*(1, 26) = 7.90, *p* = 0.009, $\eta ^2_p$ = 0.23. Participants’ preference for S_1_-repetitions over S_1_-switches was larger when the previous mode was free (90.5% vs. 32.1%) than when the previous mode was forced (84.7% vs. 39.6%). Furthermore, there was a significant 2-way interaction between SOA and S_1_ transition type, *F*(1, 26) = 36.76, *p* < 0.001, $\eta ^2_p$ = 0.59. As in Experiment 1, the preference to select S_1_ at long compared to short SOA was larger when S_1_ was associated with switching (22.4% vs. 49.3%) as compared to repeating tasks (84.2% vs. 91.0%). The 2-way interaction between SOA and previous mode (*p* = 0.390, $\eta ^2_p$ = 0.03) and the three-way interaction (*p* = 0.689, $\eta ^2_p$ = 0.01) were not significant.

### Discussion

The results of this experiment replicate all major findings of Experiment 1 and hence extend these findings to an environment with only forced no-go and free-choice trials (i.e., no forced-single trials as in Experiment 1). Specifically, we once again observed PRP-like effects and switch costs, and the causes of these two different types of multitask interference appeared to interact, as the PRP-like effect was larger when switching compared to repeating tasks. Moreover, we again observed a strong influence of the previous processing mode, as reflected in longer forced no-go processing times when the previous mode was free than forced, and as also reflected in a bias to particularly prefer task repetition when the previous mode was free rather than forced. Furthermore, participants were once again biased to favor task repetitions and tasks associated with the first stimulus over the second, suggesting that other cognitive and environmental factors also influenced choice behavior.

## General discussion

In the present study, we conducted two experiments that introduced a novel “choice/no-go” multitasking paradigm. A distinctive aspect of our paradigm is that the task processing itself determines the task to be performed, in contrast to previous studies where additional instructions or changes in the environment (e.g., presenting cues or only one stimulus) are needed to dictate the required task. Specifically, in the present experiments participants were required to respond to one of two task stimuli, with the forced-no-go trials involving only one task stimulus demanding an overt response, whereas the other task stimulus was associated with a no-go response. The present findings revealed evidence of task-specific processing of the no-go task, suggesting that participants flexibly adapted to the required task by processing information of both tasks in at least some no-go trials. Notably, this approach enabled us to simultaneously investigate various forms of multitasking interference typically examined in separate paradigms (i.e., task-switching and dual-tasking paradigms). Within our General Discussion, we consider the implications of our results for research focused on understanding the causes of multitasking decrements. Furthermore, we elaborate on the factors influencing task choice behavior in free-choice trials.

### Implications for task processing in multitasking

To begin with, the present experiments demonstrate the involvement of limited-capacity processing of no-go stimuli in determining the required response. This is shown by the finding that responses in forced-no-go trials, where S_2_ is the go-stimulus, are faster at long SOA compared to short ones. This finding is analogous to the PRP effect in dual-task studies and is typically seen as a marker of cognitive limitations when central decision-making processes are required simultaneously (Ruthruff et al., 2001, Pashler, 1994, Miller & Durst, 2015,). Specifically, that PRP effect is usually explained by the central processing of S_2_-go having to wait for S_1_-central (in this case: no-go) processing to be completed (cf. Pashler, 1994, i.e., central bottleneck models) or by S_2_-go central processing operating in parallel with T1 but only receiving a small portion of cognitive resources (if any) during central S_1_-processing (cf. Navon & Miller, 2002, Mittelstädt et al., 2022b, i.e., resource models). Notably, the present PRP-like effect with no-go trials provides arguably stronger evidence for central interference than the PRP effect typically measured with two overt responses in classical PRP studies, since the latter effect might (at least partially) reflect motor interference (e.g., Jentzsch et al., 2007, Klapp et al., 2019, Ulrich et al., 2007, Bratzke et al., 2009).

Moreover, based on the comparison of S_1_-go processing in forced-no-go versus forced-single trials in Experiment 1, the present study provides evidence that favors resource sharing over bottleneck models. According to bottleneck accounts, performance in both types of forced-choice trials should be similar since the presence or absence of S_2_ should not affect processing. However, according to resource sharing accounts, performance in S_1_ processing may suffer under the presence of a no-go second stimulus, as this stimulus may draw some of the limited processing resources away from processing S_1_ in parallel—exactly as was observed in the present study (Mittelstädt & Miller, 2017, see also).

In addition to dual-task costs (e.g., PRP-like effect), the present findings also demonstrate that switch costs can be measured in forced-no-go trials. Interestingly, the PRP-like effect was larger for repetition compared to switch trials, which, in turn, provides further support for an overlap of the control processes underlying switch costs and the PRP-like effect (e.g., Band & Van Nes, 2006, Lien et al., 2003). As discussed earlier, while this pattern may be conceptualized within both resource sharing and bottleneck accounts, it seems more naturally explained by the former. Specifically, this suggests that the degree of parallel processing of S_2_ is reduced or that processing is potentially even completely serial when S_2_ involves switching, in contrast to processing when the task is repeated relative to the previous response. Thus, it appears that switch-related reconfiguration processes limit the extent of engagement in parallel processing.

By intermixing forced and free task-choice trials, the present study also allowed for an investigation into the influence of the preceding mode on subsequent forced-no-go processing. Interestingly, the study demonstrates greater switch costs in forced-choice processing of the current trial when it follows free-choice processing, as compared to when it follows processing of a forced task. One potential explanation for this observation is that participants may represent a freely chosen task more strongly than a forced-chosen task. Consequently, additional inhibitory processes might be required to disengage from a freely chosen task, compared to a forced task, when transitioning to another task—an explanation that seems to align with the task choice findings that are elaborated in the next section. A related possibility is that free- and forced-choice task goals are represented internally somewhat differently, and thus switching between these different processing modes produces switch costs, similar to when switching between different tasks. Indeed, as can be seen in "Appendix B", there were also costs in free-choice performance when the preceding mode was forced rather than free. Notably, the observation of costs when switching (free-forced and forced-free) versus repeating processing modes (free-free and forced-forced) is consistent with a recent study by Qiao et al. (2023). However, since separate cues were used in their study to indicate the required processing modes, their pattern might also reflect cue transition costs. In this context, it should also be emphasized that we observed this pattern not only in Experiment 1 but also in Experiment 2, where no forced-single trials were employed. Therefore, alterations in the environment (i.e., one versus two stimuli) cannot account for the current modulation of switch costs, offering more direct support for the notion that distinctions between free and forced task goals stem from differences in their internal representations.

### Implications for task choice in multitasking

The present study also provides new insights into the factors and mechanisms underpinning free-choice behavior. To begin with, participants were biased towards selecting the task associated with S_1_ over S_2_, and this preference increased with a larger SOA. Given that the SOA varied randomly across trials, this indicates that people reactively adjusted their task-choice behavior during a trial (c.f., Mittelstädt, Mackenzie, Braun, & ArringtonS, in press; Mittelstädt, Miller, & Kiesel 2022c). This finding extends previous studies by providing more direct evidence for the idea of a race of task-specific information processing in free-choice trials before actually selecting a task. For example, previous task-switching studies (e.g., Arrington, 2008) and dual-task studies (e.g., Kübler et al., 2018, Strobach et al., 2018) have instructed participants to randomly select a task or to randomly choose the order of responding to two tasks. Therefore, it is possible that in those studies participants chose their tasks based on stimulus order to aid randomness, which is a reasonable strategy given the randomized SOA. Relatedly, in the study by Sigman and Dehaene (2006), the bias towards selecting the task with the first-presented stimulus could merely reflect participants’ compliance with the instruction to respond to the tasks in the order they were presented. Furthermore, in dual-task studies with a voluntary task order, two overt responses are required (e.g., Kübler et al., 2018, Strobach et al., 2018). Participants may have also adopted additional strategies to coordinate two motor responses (e.g., response grouping). While response grouping strategies have sometimes been controlled in previous studies (e.g., Leonhardet al., 2011), avoiding the overlap of two motor responses may still be (at least partially) responsible for driving participants towards selecting a response as soon as information for one of the two tasks is available.

Moreover, in contrast to those previous studies, we also directly examined whether S_1_ (or S_2_) was associated with switching or repeating tasks. Our analyses revealed that participants particularly preferred selecting S_1_ when this stimulus was linked to repeating tasks compared to switching tasks. This demonstrates that cognitive factors (reflected in a task repetition bias) and environmental factors (S_1_ bias) interact to drive voluntary task-switching behavior. Specifically, considering that switching tasks require lengthier central processing, it seems reasonable for participants to engage in central processing of S_2_ if S_2_ allows them to repeat tasks, especially when SOA is short, thereby optimizing their behavior. This also aligns with a previous dual-task study, where the bias to respond to the first presented task before the second could be overridden when the first task was more difficult to process (Leonhard et al., 2011).

Interestingly, we also found that participants exhibited a particular bias toward task repetition when the preceding trial allowed a free task choice, in comparison to when it was forced. Current models of task choice behavior essentially assume that participants opt for the task with the highest representation strength (e.g., Arrington, 2008,Mittelstädt et al., 2019, Dreisbach & Fröber,^2019^). Thus, this finding may suggest that free-choice task goals held a stronger representation than forced task goals. Since we did not employ any cues to indicate which forced task to process, as was done in a prior study (Qiao et al., 2023), this bias cannot be explained by participants specifically avoiding the cognitive effort associated with processing forced-choice task cues. In the current study, however, it would have been possible for participants to avoid selecting a task that required a no-go response in the previous trial, as they might have perceived processing this task as wasted effort. Intriguingly, this should have actually resulted in the opposite pattern—a stronger preference for avoiding switching after forced-choice tasks compared to free-choice tasks— since the previous no-go task was associated with switching in the current trial. Thus, one might also speculate that participants switch more after forced-no-go trials because they actually want to complete an unfinished task (Zeigarnik, 1938), assuming that the no-go decision leaves the task in a somewhat unfinished state. Another, not mutually exclusive, possibility is that participants have an overall preference for one of the two tasks and, as a result, tend to select that task more frequently in free-choice trials. When they have two consecutive free-choice trials, they can opt for the same task both times due to their overarching preference for it. However, when the preceding trial was a forced-choice task, it could have been either the preferred or the less preferred task, so choosing the preferred task in the next trial would result in a lower proportion of repetitions.^2^

In any case, it is also useful to consider that the effects on task choice behavior were largely consistent in Experiment 2, where two stimuli were presented in both free-choice and forced-choice trials. Specifically, in addition to free-choice trials, we exclusively used forced-no-go trials instead of both forced-single and forced-no-go trials in Experiment 2. When intermixing forced-single trials as in Experiment 1 or when including free-choice trials with very long SOAs (i.e., 1000 ms, cf., Mittelstädt et al., in press), participants may have been biased to increasingly select S_1_ at long compared to short SOAs because they assumed that this would be the only stimulus or that the other stimulus would only appear after a long time. Thus, by using a pure version of the choice-no-go paradigm without single-stimulus trials and using only small temporal differences, we provide more direct evidence for a race of task-specific information in increasing goal activations of the two task goals in parallel.

Moreover, the preference for repeating free-choice tasks over forced-choice tasks cannot solely be attributed to the presence of two stimuli in the free-choice trials, unlike in hybrid task switching paradigms where only forced-single tasks are mixed with free-choice tasks. For example, it seems plausible that during free-choice trials with two presented stimuli, some parallel processing takes place, leading to additional (bottom-up triggered) between-task interference. Consequently, successful task performance in free-choice trials may require the implementation of additional control processes to shield the processing of the chosen task from the irrelevant one (e.g., intensifying focus on the chosen task and thus enhancing its activation). Thus, a bias to favor the repetition of free- over forced-choice tasks with forced-single tasks could conceivably arise from heightened task-set activation aimed at reducing between-task interference in free-choice trials. In the present study, however, some dual-task processing occurred in both forced-no-go and free-choice trials, and yet we still observed a stronger preference for task repetition after free-choice compared to forced-choice trials. Therefore, it appears more plausible that the act of choosing a task itself results in a heightened engagement with that particular task which in turn drives participants more strongly to select the same task again, compared to when the task is externally assigned. Another possibility, not mutually exclusive, is that participants might have been particularly inclined toward selecting the task linked with a no-go stimulus (leading to more frequent task switching after forced-no-go rather than free-choice tasks), as they might regard it as an unfulfilled goal that they now have the opportunity to fulfill (cf., Converseet 2023) . Finally, regardless of the specific causes of a free-choice task bias, the present study provides no evidence that the previous processing mode additionally modulates the effects of stimulus availability on task choice behavior. Thus, it does not seem that participants are less susceptible to external influences in terms of the SOA manipulation after a free task choice than after a forced task choice.

## Conclusion and Outlook

In the present study, we introduced a new “choice/no-go” multitasking paradigm to investigate whether several multitasking phenomena when dealing with multiple tasks (switch costs, dual-task costs, avoidance of task switches) can be measured within a single setting while considering shortcomings of previous paradigms (e.g., the use of task cues to indicate which task to perform in forced-choice trials, and the use of randomness instructions to constrain which task to select in free-choice trials). The empirical results across two experiments revealed that these different markers of multitasking interference can be generalized to this new paradigm and appear to be at least partially related to each other (e.g., a larger PRP-like effect for switch compared to repetition trials, a preference to process and choose the first of two task stimuli in forced- and free-choice tasks). These findings suggest overlapping underlying causes, such as the possibility that parallel task processing might be limited by switch-related reconfiguration processes or that tasks are selected with the aim of improving task performance. More generally, it thus seems a useful methodological approach to more jointly study different empirical multitasking markers, as with the present paradigm, to work towards more integrative theoretical accounts of multitasking. One promising avenue for future research in this regard could be to rely on theoretical accounts that consider how multiple tasks are represented and coordinated in working memory to flexibly adapt behavior to different (multi-)processing demands (cf. Oberauer et al., 2013,Souza et al., 2012, Hazeltine & Schumacher, 2016, Schumacher & Hazeltine, 2016, Logan & Gordon, 2001,Verschoorenetal., 2019). In doing so, it also seems useful to consider both free- and forced-choice processing demands, as the present results also provide new evidence that favors different internal representations of free- and forced-choice tasks.

## Figures and Tables

**Fig. 6:**
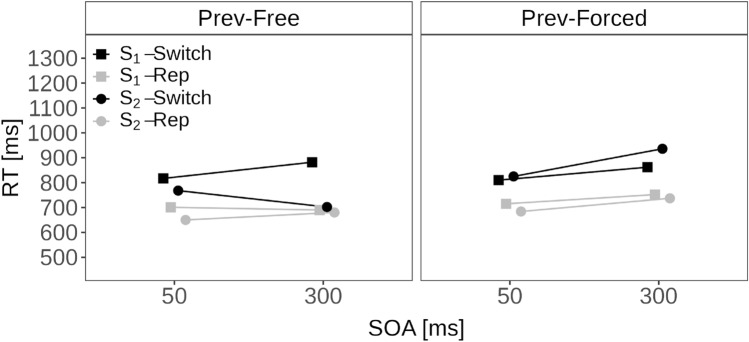
Dynamics—free: sequential effects in Experiment 1. Note. Mean reaction time (RT) for free-choice trials in a current trial as a function of the selected task (S_1_-task vs. S_2_-task), SOA (short, long) and task transition (repetition vs. switch) separately for trials preceded by free-choice trials (left column) and forced-choice trials (right column)

**Fig. 7:**
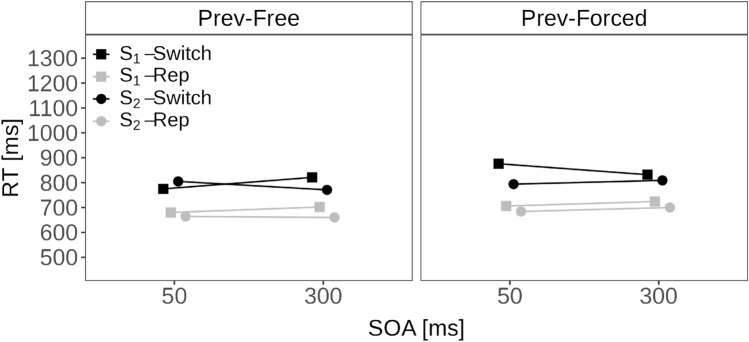
Dynamics—free: Sequential Effects in Experiment 2. Note. Mean reaction time (RT) for free-choice trials in a current trial as a function of the selected task (S_1_-task vs. S_2_-task), SOA (short, long), and task transition (repetition vs. switch), separately for trials preceded by free-choice trials (left column) and forced-choice trials (right column)

## Data Availability

Preregistrations and raw data for Experiment 1 and 2 are available via the Open Science Framework at https://osf.io/kwv92/. Materials for the experiments reported here are available from the authors upon request.

## Appendix A

### Task performance analyses of free-choice trials in Experiment 1

The interpretation of RT and PE effects in free-choice trials is complicated somewhat by the fact that participants choose which task to respond to in these trials. For example, they may be especially biased toward task repetitions when SOA is short, but especially biased toward responding to S_1_ when SOA is long. Nonetheless, at a purely empirical level it seems worthwhile to determine which experimental factors affect the mean RTs of these freely-chosen responses.

### Dynamics, free-choice trials

A 2x2 ANOVA with the factors of which task was selected (i.e., task associated with S_1_ [S_1_-task] vs. task associated with S_2_ [S_2_-task]) and SOA (short vs. long) was conducted on mean RT. There was a significant main effect of task indicating slower RTs for the S_1_-task than the S_2_-task (756 vs. 716 ms), *F*(1, 33) = 32.90, *p* < 0.001, $\eta ^2_p$ = 0.50. The main effect of SOA was also significant, reflecting slower RTs at long compared to short SOAs (754 vs. 717 ms), *F*(1, 33) = 16.39, *p* < 0.001, $\eta ^2_p$ = 0.33. The interaction was not significant (*p* = 0.387, $\eta ^2_p$ = 0.02). Descriptively, participants were faster at short compared to long SOA for trials in which they selected both the S_1_-task (732 vs. 780 ms) and the S_2_-task (702 vs. 729 ms).

A parallel ANOVA on mean PE revealed that all effects were significant. The main effect of task reflected fewer errors for the S_1_-task than the S_2_-task (2.7% vs. 6.3%) *F*(1, 33) = 14.69, *p* = 0.001, $\eta ^2_p$ = 0.31. The main effect of SOA reflected fewer errors at short compared to long SOA (3.5% vs. 5.5%), *F*(1, 33) = 7.56, *p* = 0.010, $\eta ^2_p$ = 0.19. The interaction reflected a tendency for participants to make more errors at the short than long SOA when they selected the S_1_-task (3.2% vs. 2.1%), but fewer errors at the short than long SOA when they selected the S_2_-task (3.8% vs. 8.8%), *F*(1, 33) = 12.93, *p* = 0.001, $\eta ^2_p$ = 0.28.

### Dynamics, free-choice trials, sequential effects

For free-choice trials, it was initially not possible to conduct a 4-way ANOVA with the factors of task (i.e., task associated with S_1_ [S_1_-task] vs. task associated with S_2_ [S_2_-task]), SOA (short, long), previous mode (free, forced), and task transition (repetition, switch) due to empty cells in some conditions for some participants. To address this issue, we analyzed the RT data using the percentile rank pooling method (Miller, 2021). For this analysis, each RT of each participant is converted to a percentile rank score (0–100%) relative to that individual participant’s full distribution of RTs (separately for the free and forced-choice conditions). Following Miller (2021), we then converted the RT percentile ranks back to actual RTs (R_conv_) based on an assumed standard ex-Gaussian distribution for each of the two conditions. The R_conv_ were then subjected to a 4-factor ANOVA pooling the trials of all participants within each of the 2^4^ conditions and effectively treating the trials rather than the participants as the individual random units. Similarly, we ignored random participant differences and treated the trials as random units for analyses of PEs.^3^. Figure 6 shows the corresponding mean RTs. The ANOVA revealed a significant main effect of transition, indicating faster RTs for repetition than switch trials (701 vs. 825 ms), *F*(1, 8830) = 97.83, *p* < 0.001. The main effect of task was also significant, indicating slower RTs for the S_1_-task than S_2_-task (779 vs. 748 ms), *F*(1, 8830) = 6.02, *p* = 0.014. The main effect of SOA was also significant, reflecting slower RTs at long compared to short SOA (780 vs. 746 ms), *F*(1, 8830) = 7.37, *p* = 0.007. Furthermore, there was a significant main effect of previous mode indicating slower RTs when the previous mode was forced than free (790 vs. 736 ms), *F*(1, 8830) = 18.37, *p* < 0.001. The 2-way interaction between SOA and previous mode was significant, *F*(1, 8830) = 5.52, *p* = 0.019. This interaction indicated that responses were particularly slower at long than short SOA when the previous mode was forced (822 vs. 758 ms) than free (739 vs. 734 ms). There was also a significant 2-way interaction between task and previous mode, *F*(1, 8830) = 11.01, *p* = 0.001. Responses were slower for the S_1_-task than S_2_-task when the previous mode was free (773 vs. 699 ms), whereas responses were faster for the S_1_-task than S_2_-task when the previous mode was forced (793 vs. 785 ms). The 3-way interaction between task, transition and previous mode was also significant, *F*(1, 8830) = 9.00, *p* = 0.003. This interaction indicated that switch costs for the S_1_-task were larger when the previous mode was free than forced, whereas switch costs for the S_2_-task were larger when the previous mode was forced than free. This pattern was especially evident during long SOAs, as indicated by a significant 4-way interaction. *F*(1, 8830) = 4.05, *p* = 0.032.

The most dramatic aspect of the results shown in Fig. 6, which can also be seen in the results of Experiment 2 (Fig. 7), is the strong interaction between SOA and task (S_1_ vs. S_2_) in switch trials that follow free-choice trials. When participants could choose the task freely in two successive trials (i.e., there were go stimuli for both tasks in both trials) and switched tasks for the second trial, they were slower in responding to S_1_ at the longer SOA than at the shorter one, whereas they were faster in responding to S_2_ at the longer SOA than at the shorter one. In the S_1_-switch case, S_2_ was the stimulus for the task they had just performed, and they may have had a stronger tendency to wait for this task before making the more cognitively-demanding switch; this waiting would increase RT especially when SOA was long. In the S_2_-switch case, S_1_ was the stimulus for the task they had just performed. For some reason they decided that they did not want to respond to this task, and they would have had more time to make that decision and prepare for S_2_ when SOA was longer, reducing RT to S_2_ as in the PRP-like effect.

## Appendix B

### Task performance analyses of free-choice trials in Experiment 2

This appendix reports analyses of RT and PE in the free-choice trials of Experiment 2, analogous to the analyses of Experiment 1 reported in "Appendix A".

### Dynamics, free-choice trials

A 2x2 ANOVA with the factor of which task was selected (i.e., task associated with S_1_ [S_1_-task] vs. task associated with S_2_ [S_2_-task]) and SOA (short vs. long) was conducted on mean RT. There were no significant effects (all *p* > 0.106, $\eta ^2_p$ < 0.11). Descriptively, participants were faster at short compared to long SOA for trials in which they selected the S_1_-task (732 vs. 767 ms), but slightly slower at short compared to long SOA for trials in which they selected the S_2_-task (724 vs. 719 ms).

A parallel ANOVA on mean PE revealed a significant main effect of task reflecting fewer errors for the S_1_-task than S_2_-task (2.5% vs. 5.0%) *F*(1, 26) = 10.32, *p* = 0.003, $\eta ^2_p$ = 0.28. The main effect of SOA was not significant (*p* = 0.121, $\eta ^2_p$ = 0.09). A significant interaction indicated that participants made more errors at short than long SOA when they selected the S_1_-task (3.2% vs. 1.9%), but fewer errors at short than long SOA when they selected the S_2_-task (3.2% vs. 6.8%), *F*(1, 26) = 12.39, *p* = 0.002, $\eta ^2_p$ = 0.32.

### Dynamics, free trials, sequential effects

The RT data were again analyzed using the percentile rank pooling method (Miller, 2021). Figure 7 shows the corresponding mean RTs. The 4-way ANOVA with the factors task (i.e., task associated with S_1_ [S_1_-task] vs. task associated with S_2_ [S_2_-task]), SOA (short, long), previous mode (free, forced), and task transition (repetition, switch) revealed a significant main effect of transition, indicating faster RTs for repetition than switch trials (690 vs. 810 ms), *F*(1, 7007) = 92.61, *p* < 0.001. The main effect of task was also significant, indicating slower RTs for the S_1_-task than S_2_-task (764 vs. 736 ms), *F*(1, 7007) = 5.27, *p* = 0.022. Finally, there was a significant main effect of previous mode, indicating slower RTs when the previous mode was forced rather than free (766 vs. 735 ms),*F*(1, 7007) = 6.22, *p* = 0.013. No other effects were significant (all *p*s > 0.100).

## Footnotes

1 Qualitatively similar patterns of results were also obtained when conducting the analyses separately for the first and second half of blocks (excluding practice) in the two experiments. We also directly investigated the effect of practice on RT switch costs by conducting an ANOVA with the factors of block (blocks 3–8) and task transition (switch, repetition) on mean RTs for both Experiments 1 and 2. While RTs generally decreased across blocks in both experiments, as indicated by significant main effects of block (both with *p*s < 0.022 and both $\eta ^2_p$s > 0.09), there was no evidence for differences in switch costs, as the interactions were not significant (both with *p*s > 0.265 and both $\eta ^2_p$s < 0.05).

2 To explore this possibility, we calculated the overall percentage of participants selecting either the number or letter task in free-choice trials. Subsequently, we classified each participant as either having an “overall letter preference” or an “overall number preference.” Next, we categorized the response task in each trial as either the “preferred task” or the “nonpreferred task.” In Experiment 1, when the previous task was the nonpreferred one, participants were more likely to repeat after a free-choice trial than after a forced one (63% vs. 52%, p < 0.001), but this difference was reduced, albeit still significant (p = 0.018), when the previous task was the preferred one (85% vs. 81%). In Experiment 2, when the previous task was the nonpreferred one, participants were more likely to repeat after a free-choice trial than after a forced one (67% vs. 60%, p < .001), whereas there was no significant difference when the previous task was the preferred one (84% vs. 84%, p = 0.943). Thus, these analyses suggest that the stronger repetition bias following a free-choice trial may indeed be due at least partially to an overall preference for one task, but it does not appear to fully explain this bias (e.g., when the previous task was nonpreferred, participants were still differentially biased depending on whether the previous mode was free or forced in both experiments). Note, however, that these exploratory analyses cannot completely rule out a task preference account, as it assumes that the participant’s preferred task remains stable over time, whereas it could, of course, vary over the course of the experiment. Thus, in future studies, it may be interesting to more directly investigate the influence of stable and dynamic task preferences on the present free task repetition bias (see e.g., Broeker et al., 2018, for some discussion of why and how individuals prioritize tasks).
